# Mucoadhesive Budesonide Formulation for the Treatment of Eosinophilic Esophagitis

**DOI:** 10.3390/pharmaceutics12030211

**Published:** 2020-03-01

**Authors:** Antonella Casiraghi, Chiara Grazia Gennari, Umberto Maria Musazzi, Marco Aldo Ortenzi, Susanna Bordignon, Paola Minghetti

**Affiliations:** 1 Department of Pharmaceutical Sciences, Università degli Studi di Milano, Via G. Colombo 71-20133 Milan, Italy; chiara.gennari@unimi.it (C.G.G.); umberto.musazzi@unimi.it (U.M.M.); paola.minghetti@unimi.it (P.M.); 2 CRC Materiali Polimerici (LaMPo), Department of Chemistry, Università degli Studi di Milano, Via Golgi 19-20133 Milan, Italy; marco.ortenzi@unimi.it; 3 Department of Chemistry, Università degli Studi di Milano, Via Golgi 19-20133 Milan, Italy; 4 Student of Specialization School in Pharmacy, Department of Pharmaceutical Sciences, Università degli Studi di Milano, Via G. Colombo 71-20133 Milan, Italy; susanna.bordignon@gmail.com

**Keywords:** eosinophilic esophagitis, budesonide, xanthan gum, guar gum, mucoadhesion, esophagus permeability, rheological characterization, pediatric medicine, compounded preparation

## Abstract

Eosinophilic esophagitis (EE) is a chronic immune/antigen-mediated esophageal inflammatory disease for which off-label topical corticosteroids (e.g., budesonide) are widely used in clinic. In general, thickening excipients are mixed with industrial products to improve the residence time of the drug on the esophageal mucosa. The compounding procedures are empirical and the composition is not supported by real physicochemical and technological characterization. The current study aimed to propose a standardized budesonide oral formulation intended to improve the resistance time of the drug on the esophageal mucosa for EE treatment. Different placebo and drug-loaded (0.025% *w/w*) formulations were prepared by changing the percentage of xanthan gum alone or in ratio 1:1 with guar gum. Both excipients were added in the composition for their mucoadhesive properties. The formulative space was rationalized based on the drug physicochemical stability and the main critical quality attributes of the formulation, e.g., rheological properties, syringeability, mucoadhesiveness and in vitro penetration of budesonide in porcine esophageal tissue. The obtained results demonstrated that gums allowed a prolonged residence time. However, the concentration of the mucoadhesive polymer has to be rationalized appropriately to permit the syringeability of the formulation and, therefore, easy dosing by the patient/caregiver.

## 1. Introduction

Eosinophilic esophagitis (EE) is a chronic immune/antigen-mediated esophageal inflammatory disease associated with esophageal dysfunction resulting from severe eosinophil-predominant inflammation [1,2]. EE treatment is mainly based on dietary and pharmacological interventions. The diet of patients having EE is a highly restricted regimen based on the elimination of specific allergen components for a limited number of weeks. Removal of harmful food allergens results in clinical remission of EE. After the elimination period, foods can be reintroduced in the diet sequentially in order to identify food triggers of esophageal eosinophilia and to establish a less restrictive, long-term, therapeutic diet for the effective disease management [3]. Elemental formulas and other types of elimination diets are safe and efficacious approaches for EE treatment [4], but the severe restrictions markedly reduce patient compliance [5]. The main drawbacks are the scant palatability of the highly restricted diet regimen, the marked weight loss and the high costs for the patient [6]. Therefore, pharmacological treatments are often necessary. First line therapy is based on oral proton pump inhibitors (PPIs) [7], which are effective, safe and not so expensive for the patients. If the patient does not respond to PPIs, corticosteroids are useful alternative therapeutics, since they can inhibit maturation and activation of eosinophils through suppression of the release of their stimulating cytokines. Inhaled budesonide (BU) and fluticasone are the most investigated drugs of this class [8,9,10]. However, the benefit-risk balance of such medicinal products may be affected by significant secondary side effects due to systemic drug absorption after pulmonary administration. It may also be affected by a low patient adherence, due to the complexity of the administration devices (e.g., metered-dose inhaler). Therefore, the interest in developing topically applied therapeutics is increasing [11,12]. In this context, off-label topical corticosteroids are frequently used in clinic: patients are trained to swallow asthma medicinal products designed initially to be inhaled or viscous oral formulations extemporaneously compounded in pharmacies. Recently, European regulatory authorities have authorized an orodispersible tablet loaded with BU indicated for the treatment of EE in adults older than 18 years of age. This medicinal product is not suitable for pediatric patients since they require adjustments in strength and dosage form in comparison to adults [13,14,15]. 

When an authorized medicinal product is not available on the market, the compounding of extemporaneous preparations by the community and hospital pharmacists is crucial to meet the special needs of patients [16,17]. The compounding activities should be based on the provisions of the Good Compounding Practice and other available technical guidelines to assure the required quality of the magistral preparation [18]. 

In the case of the BU for EE, a certain number of studies in the literature suggested the clinical efficacy of viscous preparations. Frequently, they have been prepared by mixing a commercial sterile suspension of BU to be nebulized, which is indicated in the treatment of bronchial asthma and in infants and children with croup, with a thickening agent (e.g., sucralose) [19,20,21]. Alternatively, cellulose derivatives [22] or gums [23,24] have also been added. Hefner et al. [25] compared the technological performances of different types of thickening agents like sucralose, xanthan gum or honey, demonstrating that the gum permitted a better residence time of the active pharmaceutical ingredients (API) on the esophageal mucosa. However, such pieces of evidence have been obtained using a medicinal product as an API source instead of the API. Although such an approach seems practical to meet patient’s needs, the use of a medicinal product can determine the presence of unnecessary excipients in the final preparation and can cause problems of physical compatibility to arise with all the adopted substances. Moreover, the exact quantitative composition of the medicinal product is generally unknown. In this light, it is preferable that compounding starts from the raw materials (pure active principle and excipients). Alternatively, a proprietary excipient mixture can be used as a formulation base (e.g., Mucolox™) [26].

The development of standardized formulations can be a valid strategy to support pharmacists in their activities, reducing heterogenicity and uncertainty in compounding procedures, improving the quality of the final preparation, at the same time. Indeed, the availability of well set up and validated operating procedures is instrumental in assuring the quality of the magistral preparation.

The aim of this work is to propose a standardized BU oral formulation to improve the residence time of the drug on the esophageal mucosa. Starting from a formulation already in use in hospital pharmacies, based on xanthan gum, six pharmacists were enrolled in the compounding process to verify its reproducibility. On the bases of these results, the formulation and the compounding procedures were optimized. Considering the evidence, reported in the literature, on the combined use with galactomannans, guar gum was selected to compare its performances, when in ratio 1:1, as opposed to xanthan gum alone. The formulative space was rationalized based on the drug’s physicochemical stability and the main critical quality attributes of the formulation, such as rheological properties, mucoadhesiveness and in vitro penetration of BU in porcine esophageal tissue. 

## 2. Materials and Methods 

### 2.1. Materials 

Micronized Budesonide (BU) was obtained from Farmabios, Gropello Cairoli, PV, Italy. Guar Gum (GG, viscosity min 5000 mPas) was kindly gifted by Lamberti spa, Albizzate, VA, Italy. All other materials were obtained from the named supplier: Xanthan Gum (XG, viscosity more than 1200 mPas), (ethylenedinitrilo)tetraacetic acid disodium salt dihydrate (EDTA), sodium benzoate, sodium saccharin (Farmalabor, Canosa di Puglia, BAT, Italy), glycerin, sodium dihydrogen phosphate, orthophosphoric acid, ethanol chemical grade (VWR International, Milan, Italy).

Acetonitrile was HPLC-gradient grade. Purified water was obtained from the purification system Milli-Q, according to Ph. Eur. 10.0 ed.

### 2.2. Preparation of Oral Formulations

The exact amount of glycerin (23.6 mL) was weighed on an analytical balance and poured into a beaker. Sodium saccharin, EDTA and sodium benzoate were crushed to a fine powder with mortar and pestle, then, the exact amount of each was weighted and the powders were transferred into the same beaker. Then, XG or the mixture of gums (XG:GG) 1/1 *w/w* was added. All substances were mixed to form a homogeneous mixture, then, the exact weighted amount of BU (1 mg/4 mL) was added and carefully mixed. Purified water was weighed and added, then stirred until a uniform system was obtained. Different percentages of gums were used: F1P was prepared using XG 2% *w/w*, F2P with XG:GG 2% *w/w*, F3P with XG 1.5% *w/w* and F4P with XG:GG 1.5% *w/w*. Placebo formulations were prepared for rheological and technological evaluation. BU was added only to obtain final loaded formulations F1, F2 and F4. The composition of the formulations is reported in Table 1.

### 2.3. Measurements of pH Values of the Formulations

The pH was measured at time T = 0, using a pHmeter CyberScan 1100 (Eutech Instruments/Thermo Fisher Scientific, Waltham, MA, USA).

### 2.4. Drug Content

About two grams of each formulation were exactly weighed and transferred into a 10 mL amber glass volumetric flask, bringing up to volume with ethanol. Then, the flask was placed in an ultrasound bath for 20 min. The sample was then centrifuged for 5 min at 3500 rpm. A portion of the supernatant was diluted to 2:5 with the mobile phase composed of phosphate buffer pH 3.2:acetonitrile:ethanol (68:30:2 *v*/*v*/*v*) and analyzed [23]. The remaining supernatant was completely and accurately removed and 10 mL of fresh ethanol were added to repeat the same extraction procedure. For each preparation, extraction was performed twice.

### 2.5. Stability Study

Samples of F1 were stored in an incubator (INCU-Line, VWR International, Milan, Italy) at 40 °C. The drug content was measured at time T = 0, T = 10, 20, 60 days. Evaluation of BU content was also performed after T = 30 days at room temperature exposed to light.

### 2.6. Determination of Rheological Properties

The steady and dynamic shear rheological properties of the formulations were carried out using a controlled stress/strain rheometer Anton Paar MCR 302 (Anton Paar GmbH, Graz, Austria) equipped with a plate-plate geometry (25 mm diameter and 500 μm gap). The temperature was controlled by a Peltier system on the bottom plate. Each sample was transferred to the rheometer plate and was then equilibrated at 25 °C for 5 min before steady and dynamic shear rheological measurements were taken.

#### 2.6.1. Steady Shear Measurements

Flow behavior was evaluated at controlled strain mode to obtain flow rheological data (shear stress and shear rate) over a shear rate range of 0.1–100 s^−1^ at 25 °C. The shear stress-shear rate data were fitted to the well-known power law and Casson models [27] to describe the flow properties of the samples.

#### 2.6.2. Dynamic Shear Measurements

Dynamic rheological data were obtained from frequency sweeps over the range of 0.628–62.8 rad s^−1^ at 2% strain using a small-amplitude oscillatory rheological measurement. The applied 2% strain was confirmed within the linear viscoelastic region by strain sweep measurement. Frequency sweep tests were also performed at 25 °C. The RheoCompass software (Anton Paar GmbH, Graz, Austria) was used to obtain the experimental data and to calculate the storage (or elastic) modulus (G′), loss (or viscous) modulus (G″) and loss factor (tan δ = G″/G′).

### 2.7. Quantitative Determination of Syringeability

The measurement of the injection force was performed in compression mode by using a software-controlled texture analyzer (Instron 5965, ITW Test and Measurement Italia S.r.l., Trezzano sul Naviglio, Italy). A 10 mL syringe (SOFT-JECT^®^, VWR International, Milan, Italy) filled with 9 mL formulation was positioned in the dynamometer holder, downward needle. The plunger end of the syringe was placed in contact with a 50 N loading cell. Testing was carried out at the crosshead speed of 0.5 mm/s. The loading force required to displace the plunger was measured as a function of plunger displacement. The following parameters were also determined from the force-displacement plot:

- Plunger-stopper break loose force (or “initial glide force,” PBF): the force required to initiate the movement of the plunger;
- Maximum force (MF): the highest force measured before the plunger finishes its course at the front end of the syringe;
- Dynamic glide force (DGF): the force required to sustain the movement of the plunger to expel the content of the syringe.

**Figure 1:**
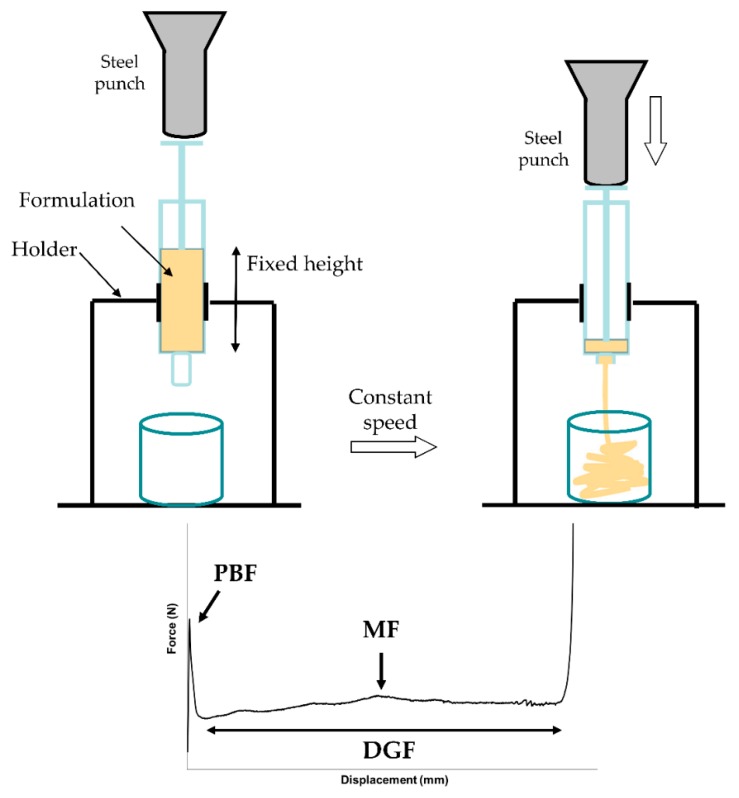
Syringeability test setting and a general force versus displacement curve (PBF: plunger-stopper break loose force; MF: Maximum force; DGF: Dynamic glide force).

A schematic representation of the syringeability test setting and a general force-displacement plot are illustrated in Figure 1. The registered force values were normalized by dividing them for the cross-sectional area of the cylindrical plunger. The experiments were performed in triplicate.

### 2.8. Mucoadhesive Properties and In Vitro Esophagus Penetration

The in vitro mucosal penetration study was performed adapting the flow-through test described by Cilurzo et al. [28] by using fresh porcine esophageal tissue obtained by a local slaughterhouse. The mucosa epithelium was separated by mucosal specimens using a scalpel.

**Figure 2:**
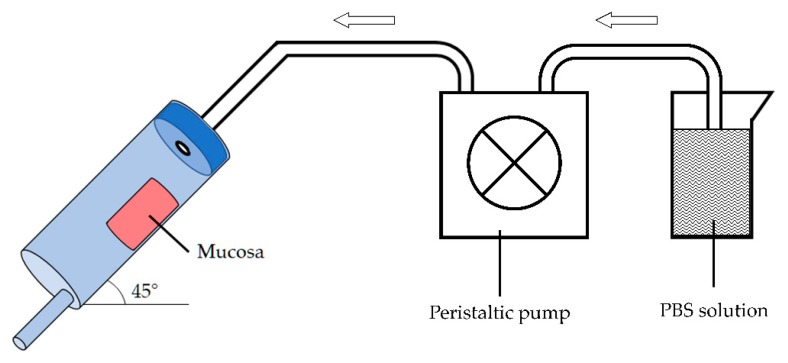
Apparatus used to evaluate mucoadhesive properties and mucosal penetration in vitro.

In-house equipment of three components was built up (Figure 2): (a) in series mucosa supports set at an angle of 45°, (b) peristaltic pump and (c) collector of fractions. The apparatus was designed to investigate at the same time the elution of the preparation from the mucosal surface and the drug penetration into the tissue.

A dose of the selected formulation was deposited onto a 3 × 2 cm mucosal surface corresponding to a total amount of about 500 mg. Then, the porcine esophageal membrane was placed on the sample support and pH 6.8 phosphate buffer solution (PBS) was dropped at a rate of 1 mL/min to simulate the physiological environment and the saliva swallowing. The test was performed at room temperature.

The residence time was qualitatively estimated by checking the time required for each formulation to be thoroughly washed away from the surface of the mucosa exposed to the washing medium. In all cases, after 30 min from the beginning of the experiment, the apparatus was dismantled, and the applied test sample was peeled away using an adhesive tape strip [28]. The mucosa samples were then washed from both sides with 5 mL of purified water and homogenized. The amount of the penetrated drug was extracted with 2 mL methanol and assayed by HPLC.

### 2.9. Drug Content Analysis

The analysis was carried out by HPLC-DAD (Agilent 1100, Agilent, Santa Clara, CA, USA) using an RP-C18 column (LC 150 × 4.6 mm, 5 µm) with pre-column (Phenomenex, Torrance, CA, USA). The mobile phase was composed of phosphate buffer pH 3.2:acetonitrile:ethanol [29]. The elution was carried out in gradient (Table 2). The flow rate was 1.7 mL/min, the wavelength was set at 240 nm and the injection volume at 10 µl. The retention times of the 22R and 22S epimers were 8.8 min and 9.1 min, respectively. A calibration curve was prepared twice solubilizing in ethanol 10 mg of BU in a 100 mL volumetric flask. Dilution to obtain 0.1, 0.2, 0.5, 1 and 2 μg/mL concentration were performed (22R: R^2^ = 0.99930; 22S: R^2^ = 0.99999). 

### 2.10. Statistical Analysis

Tests for significant differences between means were performed by the one-way ANOVA followed by Turkey-Kramer post-analyses. Differences were considered significant at the *p* < 0.05 level.

## 3. Results and Discussion

As the number of patients suffering from EE is increasing, among both adults and young population, the need to have a standardized topical dosage form has grown. Moreover, the widespread habit of mixing the industrial inhalation product with sweeteners (among them is largely used an artificial sweetener based on sucralose in maltodextrin/glucose) should not be the first choice. Instead, the use of a viscous preparation as a vehicle is well established in improving the efficacy of topical corticosteroid administration. The proprietary blend Mucolox™ gave good results [26], but it is composed of a very complex blend. For these reasons, other simpler compositions, already in use in hospital pharmacies, deserve to be investigated. Quite diffused is the use of viscous formulation containing XG as a thickening and rheological agent (F1P and F1, Table 1).

XG is a polysaccharide, largely applied in the food industry. It is highly stable in a wide range of pH and ionic strength, and, dispersed in water at moderate temperatures, it increases its viscosity even at low concentration [30,31]. Temperature preparation can modify the ordered or disordered state of xanthan chains, as this state is controlled by temperature.

According to the already in use hospital preparation (F1, Table 1), six pharmacists were enrolled and each of them compounded a batch. At the end of the preparation process, performed at room temperature, BU was completely dispersed, and the gel-like liquid appeared homogeneous. Dispersion is due to the low BU solubility in water [32]; good improvement of solubilization could be obtained using mixtures with ethanol, but this approach must be discarded in the pediatric population for limitations in the use of this excipient [33]. The use of glycerin, usually foreseen in mixture with rheological agents, can contribute in improving BU solubility, but this is not enough to obtain its complete solubilization. The measured pH and drug content are reported in Table 3. pH values were close to 5 and no significant changes in pH were observed during the storage period. To exactly measure the drug content, the extraction procedure reported in the literature [23] was performed twice, as a too high amount of BU remained not extracted after a single exposure to solvent. Variability among operators was low (CV% = 2.7) and immediately after preparation, the mean content was within the range of acceptability, fixed in ±10% *w*/*w*, according to the Italian Pharmacopoeia (Table 3). The chemical stability study showed that the preparation has good stability, as evidenced by the accelerated stability study (storage for over 60 days in an incubator at 40 °C; Table 3) and confirmed the need to avoid the exposure to light for long period when the preparation is handled by caregivers or patients. Indeed, preparations left at room temperature for over 30 days at the light showed a reduction of more than 50% in strength (Table 3). The addition of sodium benzoate allows to avoid microbiological contamination [23].

To test the technological properties of these systems, their adhesion on the site of action (i.e., esophageal tissue) or the handling during the application, F1P was modified and a mixture of XG and GG was used (F2P, Table 1). The addition of galactomannans such as locust bean gum or GG to a solution of XG at room temperature causes a synergistic increase in viscosity, both at dilute and concentrated solution [27]. GG is a galactomannan that forms colloidal solutions with elevated viscosity even at very low concentrations. It is anionic in nature and it remains stable and gives consistent viscosity over a wide pH range [34]. The viscosity of XG:GG mixtures depends on operational properties such as the dissolution temperature of gums, polymer concentration and relationship between XG and galactomannan. Indeed, with the polymer concentration and the ratio between the gums being fixed, in case of extemporaneous preparation, attention must be paid to the temperature at which each polysaccharide has been solved [35]. 

**Figure 3:**
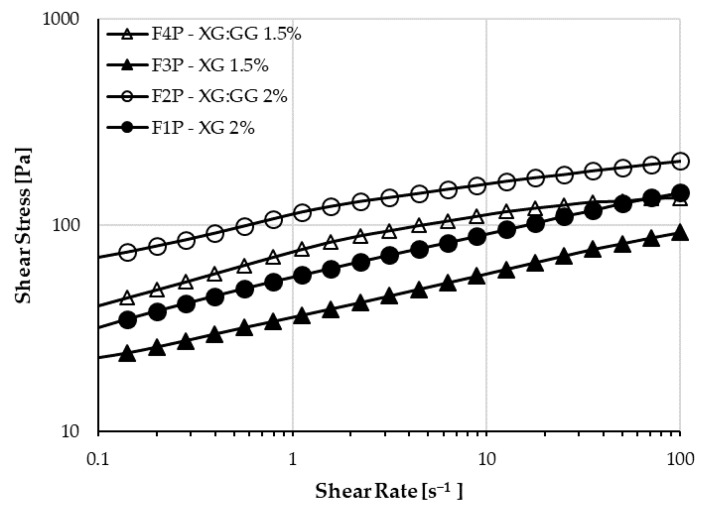
Plots of shear stresses versus shear rates of XG and XG:GG mixtures.

Rheological characterization was performed on the placebo samples based on XG (F1P and F3P) and XG:GG (F2P and F4P), mixed at room temperature. They have different behaviors: formulations containing the mixture of the gums have higher shear stress in comparison to those containing only XG, as already reported in the literature [35], but the shear sensitivity is different. As shown in Figure 3, XG samples have a linear stress-strain dependence, whereas XG:GG samples have a pseudoplastic behavior, with the stress vs. strain lowering at higher shear rates. When reaching the highest shear rates, XG:GG 1.5% (F4P) starts having lower stress than XG 2% (F1P). Given the slope of the curves (i.e., pseudoplastic vs. linear dependence), the behavior is expected to be confirmed at shear rates higher than 100 s^−1^, which is the highest that can be reached with the experimental setup used. In addition, samples loaded with the active principle were investigated and it was concluded that the behavior was not influenced by the presence of BU (F1 and F4, Table 4). The pseudo-plastic behavior of XG:GG samples indicates that physicochemical properties of such formulations are similar to the ones of linear polymers, i.e., the effect of ionic interactions or other weak bonds (such as hydrogen or van der Waals bonds) creating branching and reversible crosslinking between the macromolecular chains of the gums and other ingredients of the formulations is negligible. This behavior is in agreement with the one previously observed by Jo et al. on gum mixtures [27]. The same authors evidenced a pseudoplastic behavior also for the single gums in water solution. In our work, the presence of glycerin, adding a lot of –OH groups in the formulation, seems to increase the formation of entanglements and weak bonds in the formulation of XG alone, within the shear range tested. Table 4 also shows the difference in viscosity of the samples at the highest shear rates, confirming that the XG:GG sample viscosity has a far less marked increase than that of XG samples in the 50–100 s^−1^ range. This trend is due to the pseudoplastic behavior of XG:GG samples.

**Figure 4:**
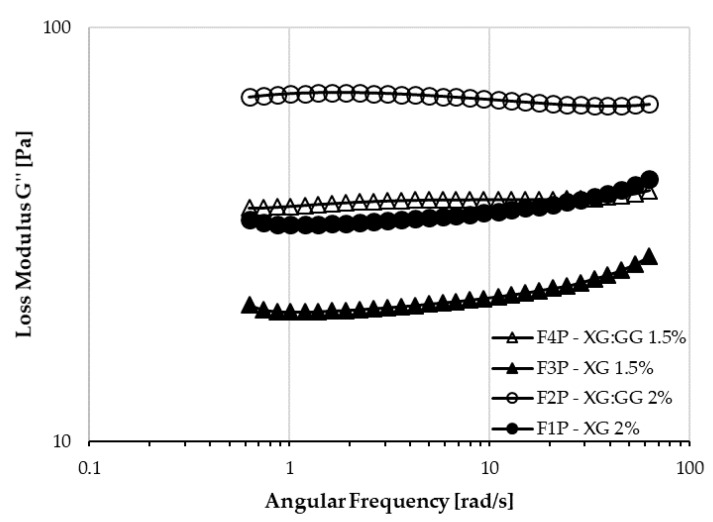
Plots of log ω versus log G″ of XG and XG:GG mixtures.

A different behavior among the formulations was also evidenced from the performed dynamic shear measurements. In particular, as shown in Figure 4, a markedly different trend in loss modulus is visible. In XG:GG samples, the G’’ has only slight variations over the angular frequency range used, whereas in XG samples, it markedly increases as the frequency gets higher, especially at the highest frequencies.

To identify elastic or viscous behavior, loss factor values can be calculated. When this value is smaller than the unit, a sample is more elastic than viscous, and this has been suggested as a rheological criterion for safe-swallow foods meant for dysphagia [36]. All proposed formulations had low loss factor values (Table 4). Moreover, while loss factor values for F1P, F2P and F3P were similar, F4P had a loss factor significantly higher than all the other samples (*p* < 0.0001).

The administration of this preparation in very young patients was performed by caregivers, normally the parents, by means of a big syringe containing 60 mL of the formulation. During this treatment, some difficulties due to the hard extrusion of the formulation from the syringe were described by the users. The values measured for the injection force measured for placebo formulations are reported in Table 4.

In the force vs. displacement plot of low-viscosity formulations (Figure 1), two different portions can be identified: the former is related to the force required to displace the plunger, (i.e., PBF). This event is followed by a plateau (second portion), indicating that the streamline of the formulation through the needle occurs with a constant force. In this portion, the average load required to sustain the movement of the plunger to expel the content of the syringe is calculated and reported as DGF. 

PBF values were not statistically different among the tested products (*p* = 0.3890), suggesting that the force required to initiate the movement of the plunger was independent from the formulation.

The measured extrusion forces showed that F4P required a slightly higher extrusion force (in terms of DGF) with respect to the other formulations, even if the differences were not statistically significant (*p* = 0.1565). Moreover, in the shear stress plots, F4P showed also a pseudoplastic behavior that could not be detected with the syringeability test, probably because the applied force felt below the useful range of stress. In the case of this pseudoplastic formulation, taking into consideration the loss factor value, another rheological parameter, we can observe that it was higher if compared to the other formulations, suggesting that F4P has a more pronounced viscous component, even if its overall behavior was mainly elastic. However, overall results show that in any case low forces are needed to extrude the formulations from a syringe, despite the differences highlighted in the rheological studies. 

The chemical and technological effects of the combination of the two gums in the revised formulations are reported in Table 5. Formulation F3 was considered of minor interest because of the lower shear stress and therefore it was not further investigated. Placebo formulations showed pH values from 4.975 (F2P) to 5.155 (F4P). As in the case of F1, the addition of BU did not change these values (Table 5). The formulations can be expected to be non-irritating to the buccal mucosa since the pH of the esophageal mucosa is reported to be 6.8 in healthy subjects [37]. 

As far as the mucoadhesive properties are concerned, comparing the formulations, a similar sliding time from the mucosa samples was observed for all of them. Thanks to the establishment of interactions between the mucosal layer and the bioadhesive polymer, it was possible to obtain formulations able to persist onto the mucosal surface for about 30 min.

The good mucoadhesive properties can be due to the presence of free –COO¯ groups of XG. As a matter of fact, it is well known that polymers that exhibit a high density of available hydrogen bonding groups (–COO^−^ groups of XG) can interact more strongly with mucin glycoprotein [38]. Moreover, hydrogen bonds are involved in the formation of a strengthened network, thus contributing to the good bioadhesive strength of the tested formulations. The permanence time onto the mucosa samples was not influenced by the presence of the drugs into the formulations. Because of the similar residence time, the percentages of BU that penetrated the mucosa by the different formulations were not significantly different (Table 3). A very small percentage of BU loaded remained in the mucosae; therefore, to optimize the use of the corticosteroid, further evaluations could be done with formulations containing a reduced amount of the active principle with respect to that used in the present work.

As it is known, penetration of BU into the mucosa is the final combination of many events; the physicochemical characteristics of the molecule, and the mucoadhesive properties and viscosity of the formulation. The advantage of increased viscosity of the formulation is a reduced outflow from the mucosal surface, with a consequent increase in the contact time of the drug with the mucous membrane, and therefore, in its penetration into the tissue. On the other hand, an excessive viscosity could obviously create problems in the extrusion from a syringe. The results showed that the use of the two gums in combination can help to modulate the viscosity of the formulation, however, remaining within an optimal range such as to be retained onto the mucosal surface for enough time. Moreover, thanks to its pseudoplastic behavior, the extrusion of such formulations through a syringe can take place in a feasible way. Unfortunately, this aspect could not be fully investigated through the experiments carried out in this work, since the viscosity data should be collected in a higher shear range to highlight a correlation between syringeability test and viscosity measurements.

## 4. Conclusions

The prepared formulations showed suitable technological and rheological characteristics for the treatment of EE. As the addition of GG to XG caused an increase in viscosity, the choice of a mixture of gums allows the use of thickening agents in a reduced amount. In this case, the obtained results were not significantly different from those measured with the formulation already in use. Considering the limited percentage of BU absorbed in the in vitro penetration experiments, further evaluations should be carried out to rationalize the drug content and reduce the risk of systemic side effects.

## Figures and Tables

**Table 1:** Composition (expressed in grams) of the formulations for 240 mL (four doses each of 60 mL).

Excipients	F1P	F1	F2P	F2	F3P	F4P	F4
Budesonide (BU)	-	0.06	-	0.06	-	-	0.06
Xanthan gum (XG)	4.80	4.80	2.40	2.40	3.60	1.80	1.80
Guar gum (GG)	-	-	2.40	2.40	-	1.80	1.80
Sodium saccharin	0.24	0.24	0.24	0.24	0.24	0.24	0.24
Glycerin	29.74	29.74	29.74	29.74	29.74	29.74	29.74
EDTA	0.24	0.24	0.24	0.24	0.24	0.24	0.24
Sodium benzoate	0.45	0.45	0.45	0.45	0.45	0.45	0.45
Water up to (mL)	240	240	240	240	240	240	240

**Table 2:** The gradient of the mobile phase.

Time (min)	Phosphate Buffer pH 3.2 (% *v/v*)	Acetonitrile (% *v/v*)	Ethanol (% *v/v*)
0–10	68→50	30→48	2
10–11	50	48	2
11–16	50→68	48→30	2

**Table 3:** Chemical characterization of the in-use preparation in the hospital pharmacy (F1: XG 2%). pH values and drug content at preparation time (T = 0) and at the end of the storage period in an incubator in dark condition (T = 60 days). Drug content determined after 30 days at room temperature (r.t.) exposed at the light.

Batch	pH		Micronized Budesonide Content (mg/mL, *n* = 6)		
	T = 0	60 days	T = 0	60 days, 40 °C, dark	30 days, r.t., light
F1	4.629 ± 0.020	4.814 ± 0.115	0.262 ± 0.007	0.246 ± 0.018	0.103 ± 0.022

**Table 4:** Viscosity values and injection force measurements of each formulation.

Form.	Viscosity (Pa*s)			Δη 100 vs. 50 s^−1^ (%)	Loss factor °	Injection force		
	50.1 (s^−1^)	70.8 (s^−1^)	100 (s^−1^)			DGF (kPa)	MF (kPa)	PBF (kPa)
F1P	128.15	136.12	144.25	12.6	0.157 ± 0.015	90.01 ± 31.16	77.87 ± 14.50	66.25 ± 5.24
F1	127.22	135.75	144.83	13.8	0.160 ± 0.016	-*	-*	-*
F2P	190.48	197.68	204.46	7.3	0.190 ± 0.040	83.28 ± 8.68	87.08 ± 7.30	58.57 ± 14.13
F3P	81.57	87.09	92.42	13.3	0.171 ± 0.018	78.08 ± 24.23	85.00 ± 29.67	63.11 ± 19.28
F4P	130.67	134.89	135.60	3.8	0.231 ± 0.045	121.69 ± 14.75	126.39 ± 14.78	89.49 ± 27.24
F4	126.77	128.5	130.02	2.6	0.242 ± 0.047	-*	-*	-*

Note: ° average ratio and standard deviation of G”/G’ over the whole curve; * value not determined.

**Table 5:** Chemical and technological characterization of the modified preparation.

Batch	pH T = 0	Permanence Time (min)	BU Penetrated the Mucosa	
			(mg/g mucosa)	(%)
F1	4.976	28 ± 4	0.790 ± 0.192	0.610 ± 0.132
F2	5.156	29 ± 2	0.783 ± 0.231	0.562 ± 0.152
F4	5.068	25 ± 5	0.901 ± 0.367	0.682 ± 0.270
